# Redetermination of tamarugite, NaAl(SO_4_)_2_·6H_2_O

**DOI:** 10.1107/S1600536813025154

**Published:** 2013-09-21

**Authors:** Kurt Mereiter

**Affiliations:** a Institute of Chemical Technology and Analytics, Vienna University of Technology, Getreidemarkt 9/164SC, A-1060 Vienna, Austria

## Abstract

The crystal structure of tamarugite [sodium aluminium bis­(sulfate) hexa­hydrate] was redetermined from a single crystal from Mina Alcaparossa, near Cerritos Bayos, southwest of Calama, Chile. In contrast to the previous work [Robinson & Fang (1969 ▶). *Am. Mineral.* **54**, 19–30], all non-H atoms were refined with anisotropic displacement parameters and H-atoms were located by difference Fourier methods and refined from X-ray diffraction data. The structure is built up from nearly regular [Al(H_2_O)_6_]^3+^ octa­hedra and infinite double-stranded chains [Na(SO_4_)_2_]^3−^ that extend parallel to [001]. The Na^+^ cation has a strongly distorted octa­hedral coordination by sulfate O atoms [Na—O = 2.2709 (11) – 2.5117 (12) Å], of which five are furnished by the chain-building sulfate group S2O_4_ and one by the non-bridging sulfate group S1O_4_. The [Na(SO_4_)_2_]^3−^ chain features an unusual centrosymmetric group formed by two NaO_6_ octa­hedra and two S2O_4_ tetra­hedra sharing five adjacent edges, one between two NaO_6_ octa­hedra and two each between the resulting double octa­hedron and two S2O_4_ tetra­hedra. These groups are then linked into a double-stranded chain *via* corner-sharing between NaO_6_ octa­hedra and S2O_4_ tetra­hedra. The S1O4 group, attached to Na in the terminal position, completes the chains. The [Al(H_2_O)_6_]^3+^ octa­hedron (〈Al—O〉 = 1.885 (11) Å) donates 12 comparatively strong hydrogen bonds (O⋯O = 2.6665 (14) – 2.7971 (15) Å) to the sulfate O atoms of three neighbouring [Na(SO_4_)_2_]^3−^ chains, helping to connect them in three dimensions, but with a prevalence parallel to (010), the cleavage plane of the mineral. Compared with the previous work on tamarugite, the bond precision of Al—O bond lengths as an example improved from 0.024 to 0.001 Å.

## Related literature

For the previous structure determination of tamarugite, see: Robinson & Fang (1969 ▶). For mineralogical data of tamarugite, see: Anthony *et al.* (2003 ▶). For the mineralogy of three sulfate deposits of northern Chile including Mina Alcaparrosa, see: Bandy (1938 ▶). For the recently described new sulfate mineral alcaparrosite, see: Kampf *et al.* (2012 ▶). For crystal structures of the related aluminium sulfate hydrates mendozite [NaAl(SO_4_)_2_·11H_2_O], sodium alum [NaAl(SO_4_)_2_·12H_2_O], alunogen [Al_2_(SO_4_)_3_·17H_2_O] and apjohnite [MnAl_2_(SO_4_)_4_·22H_2_O], see: Fang & Robinson (1972 ▶); Cromer *et al.* (1967 ▶); Menchetti & Sabelli (1974 ▶, 1976 ▶).

## Experimental

### 

#### Crystal data

NaAl(SO_4_)_2_·6H_2_O
*M* *_r_* = 350.19
Monoclinic, 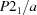

*a* = 7.3847 (3) Å
*b* = 25.2814 (15) Å
*c* = 6.1097 (3) Å
β = 94.85 (2)°
*V* = 1136.57 (10) Å^3^
*Z* = 4
Mo *K*α radiation
μ = 0.66 mm^−1^
*T* = 295 K
0.48 × 0.25 × 0.20 mm

#### Data collection

Bruker SMART CCD diffractometer
Absorption correction: multi-scan (*SADABS*; Bruker, 1999 ▶) *T*_min_ = 0.74, *T*_max_ = 0.88
17967 measured reflections
3316 independent reflections
2826 reflections with *I* > 2σ(*I*)
*R*_int_ = 0.029

#### Refinement

*R*[*F* ^2^ > 2σ(*F* ^2^)] = 0.025
*wR*(*F* ^2^) = 0.061
*S* = 1.14
3316 reflections
194 parameters
H-atom parameters constrained
Δρ_max_ = 0.48 e Å^−3^
Δρ_min_ = −0.36 e Å^−3^

### 

Data collection: *SMART* (Bruker, 1999 ▶); cell refinement: *SAINT* (Bruker, 1999 ▶); data reduction: *SAINT*; program(s) used to solve structure: *SHELXS97* (Sheldrick, 2008 ▶); program(s) used to refine structure: *SHELXL97* (Sheldrick, 2008 ▶); molecular graphics: *DIAMOND* (Brandenburg, 2012 ▶); software used to prepare material for publication: *SHELXL97* and *publCIF* (Westrip, 2010 ▶).

## Supplementary Material

Crystal structure: contains datablock(s) I, New_Global_Publ_Block. DOI: 10.1107/S1600536813025154/pj2005sup1.cif

Structure factors: contains datablock(s) I. DOI: 10.1107/S1600536813025154/pj2005Isup2.hkl

Additional supplementary materials:  crystallographic information; 3D view; checkCIF report

## Figures and Tables

**Table 1:** Selected bond lengths (Å)

Na—O6	2.2709 (11)
Na—O3	2.3389 (12)
Na—O8^i^	2.4153 (12)
Na—O5^ii^	2.4814 (12)
Na—O7^ii^	2.5020 (11)
Na—O7^i^	2.5117 (12)
Al—O10*W*	1.8755 (10)
Al—O13*W*	1.8776 (11)
Al—O11*W*	1.8816 (10)
Al—O12*W*	1.8816 (10)
Al—O9*W*	1.8904 (10)
Al—O14*W*	1.9054 (11)
S1—O3	1.4671 (10)
S1—O2	1.4738 (10)
S1—O1	1.4759 (10)
S1—O4	1.4825 (10)
S2—O8	1.4653 (10)
S2—O6	1.4723 (10)
S2—O5	1.4814 (10)
S2—O7	1.4826 (10)

Symmetry codes: (i) 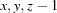; (ii) 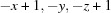.

**Table 2:** Hydrogen-bond geometry (Å, °)

*D*—H⋯*A*	*D*—H	H⋯*A*	*D*⋯*A*	*D*—H⋯*A*
O9*W*—H9*A*⋯O2^iii^	0.80	2.00	2.7971 (15)	173
O9*W*—H9*B*⋯O4^iv^	0.80	1.83	2.6282 (14)	178
O10*W*—H10*A*⋯O5^ii^	0.80	1.87	2.6665 (14)	173
O10*W*—H10*B*⋯O6^iii^	0.80	1.78	2.5697 (14)	169
O11*W*—H11*A*⋯O7	0.80	1.83	2.6315 (14)	176
O11*W*—H11*B*⋯O3	0.80	1.93	2.7236 (14)	175
O12*W*—H12*A*⋯O4^iii^	0.80	1.89	2.6787 (14)	171
O12*W*—H12*B*⋯O2^v^	0.80	1.84	2.6330 (14)	169
O13*W*—H13*A*⋯O1^vi^	0.80	1.86	2.6474 (14)	169
O13*W*—H13*B*⋯O1^vii^	0.80	1.88	2.6698 (14)	171
O14*W*—H14*A*⋯O5^viii^	0.80	2.18	2.7478 (15)	129
O14*W*—H14*B*⋯O8^iii^	0.80	1.96	2.7100 (17)	156

Symmetry codes: (ii) 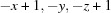; (iii) 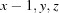; (iv) 
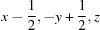
; (v) 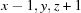; (vi) 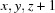; (vii) 
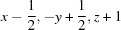
; (viii) 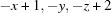.

**Fig. 1.**
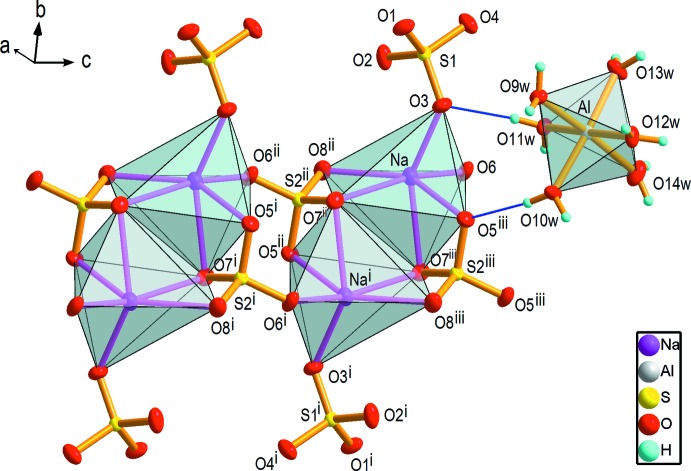
View of the two building blocks of tamarugite, the [Al(H2O)6]3+ octahedron and a segment of an infinite double-strand chain [Na(SO4)2]3- extending along [001]. Thermal displacement ellipsoids are shown at the 50% probability level. Symmetry operators: none x,y,z; (i) 1-x,-y,-z; (ii) x,y,-1+z; (iii) 1-x,-y,1-z.

**Fig. 2.**
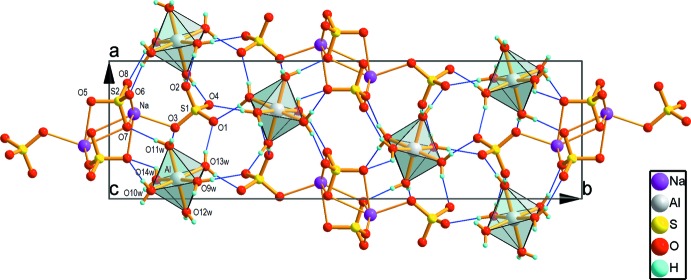
The crystal structure of tamarugite in a projection along [001], the direction of the [Na(SO4)2]3- chains. Hydrogen bonds are shown as blue lines. Only the atoms of the asymmetric unit are labeled.

**Fig. 3.**
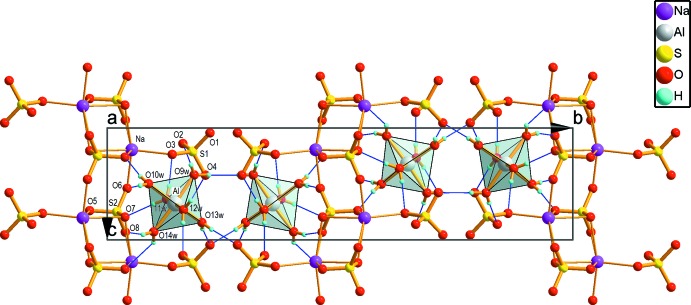
The crystal structure of tamarugite in a projection along [100]. Hydrogen bonds are shown as blue lines. Only the atoms of the asymmetric unit are labeled.

## supplementary crystallographic information

### 1. Comment

Tamarugite, NaAl(SO_4_)_2_·6H_2_O, is a secondary sulfate mineral which has been found in acidic environments generated by oxidation of sulfides like pyrite in the presence of alkali-rich aluminous rocks as Na and Al source. Classic occurrences of tamarugite are sulfate-rich weathering zones of sulfide ore deposits in the Atacama desert, Chile (Bandy, 1938). Other occurrences concern fumaroles, acid mine drainage, and burning coal dumps (Anthony *et al.*, 2003). The mineral was first described from an occurrence in the Pampa del Tamarugal, Chile, from where it inherited its name (Anthony *et al.*, 2003). The crystal structure of tamarugite was reported by Robinson & Fang (1969) as part of studies on the structural chemistry of salt hydrate minerals of Al^3+^ and Fe^3+^. Using diffraction data measured with a Buerger automated diffractometer (Weissenberg geometry, Cu Kα radiation), they obtained *R*[*F*] = 0.073 on 744 *F_hkl_* with isotropic displacement parameters. They stated that hydrogen atom positions derived from stereochemical considerations were included in this refinement, but did neither report their coordinates nor corresponding geometric parameters.

The present structure redetermination was initiated when during an examination of sulfate mineral specimens from "Alcaparossa" (Mina Alcaparrosa near Cerritos Bayos, southwest of Calama, Chile; see Bandy, 1938) colourless crystals of good quality were encountered that turned out to be tamarugite. Unit cell setting and atom positions reported by Robinson & Fang (1969) were maintained in the present study. A comparison of previous (Robinson & Fang, 1969) and present structural data of tamarugite showed a fair agreement after taking into account that e.s.d.s for atomic coordinates were previously *ca* 20 times bigger than now, where standard deviations of the Na,Al,*S*—O bond lengths are about 0.001 Å. The largest difference between the two structures was 0.044 Å for the bond S2—O5 (Table 1). The differences between previous and present non-hydrogen atom positions are 0.018 Å on average and 0.054 Å for O5.

The crystal structure of tamarugite is built up from nearly regular [Al(H_2_O)_6_]^3+^ octahedra and an infinite two-strand chain of the composition [Na(SO_4_)_2_]^3-^ extending along [001] (Fig. 1). Na is coordinated by six sulfate oxygen atoms with Na—O distances between 2.2709 (11) and 2.5117 (12) Å, mean value 2.42 Å (σ = 0.10 Å). Five of these six Na—O bonds are to the sulfate group S2O_4_ and only Na—O bond to the sulfate group S1O_4_. The coordination figure about Na can be described as a strongly distorted octahedron with *cis* bond angles between 57.26 (3) and 116.48 (4), and with *trans* bond angles between 135.96 (4) and 161.83 (5)°. This distortion is mostly due to the presence of a compact centrosymmetric group of two NaO_6_ octahedra and two S2O_4_ groups joined *via* five edge-sharing polyhedral links, one between two NaO_6_ octahedra and four between these two NaO_6_ octahedra and two adjacent SO_4_ tetrahedra (Fig. 1). Two corner-sharing links between NaO_6_ and S2O_4_ polyhedra *via* O6 expand this group into a two-stranded chain, to which at terminal position the S1O_4_ tetrahedron is attached *via* O3. The two independent sulfate groups S1O_4_ and S2O_4_ form relatively regular tetrahedra with S—O bond length in the range 1.4653 (10) – 1.4825 (10) Å and a mean bond length of S—O = 1.475 (7) Å. The O—S—O bond angles vary only over a narrow range of 3.3° (107.34 (6) – 110.61 (6)°). Six of the eight sulfate oxygen atoms are involved in two external bonds, either two hydrogen bonds (O1, O2, O4), or one Na—O and one hydrogen bond (O3, O6, O8). O5 is involved in one Na—O and two hydrogen bonds, and O7 in two Na—O and one hydrogen bonds. The [Al(H_2_O)_6_]^3+^ octahedron has a mean bond length of 〈Al—O〉 = 1.885 (11) Å, in good accord with the aluminium sulfate hydrates mendozite (NaAl(SO_4_)_2_.11H_2_O), sodium alum (NaAl(SO_4_)_2_.12H_2_O), alunogen (Al_2_(SO_4_)_3_.17H_2_O), and apjohnite (a Mn analogue of pickeringite, MgAl_2_(SO_4_)_4_.22H_2_O) (Fang & Robinson, 1972; Cromer *et al.*, 1967; Menchetti & Sabelli, 1974, 1976). It is fairly regular by having *cis* bond angles of 85.98 (5) – 93.76 (6)° and *trans* bond angles of 173.97 (5) – 177.69 (5)°. The [Al(H_2_O)_6_]^3+^ octahedron donates twelve comparatively strong hydrogen bonds with O···O = 2.6665 (14) – 2.7971 (15) Å to the sulfate oxygen atoms of three neighbouring [Na(SO_4_)_2_]^3-^ chains (Table 2). Eleven hydrogen bonds are largely linear having H···O = 1.78 – 2.00 Å and O—H···O = 156 – 178° (Table 2). Only the hydrogen bond O14W—H14A···O5^viii^ is strongly bent due to the arrangement of the acceptor oxgen atom. It has therefore an outlying geometry with H···O = 2.18 Å and O—H···O = 129°. The next nearest oxygen neighbour of H14A, O3^iii^ with H14A···O3^iii^ = 2.65 Å, is not regarded as significantly bonded and therefore was not included in Table 2. The packing diagrams shown in Figs. 2 and 3 include all hydrogen bonds of the structure. Fig. 2 shows that each [Al(H_2_O)_6_]^3+^ is hydrogen bonded with three [Na(SO_4_)_2_]^3-^ chains. Ten of the twelve different hydrogen bonds help to establish layers parallel to (010) with the composition {[Al(H_2_O)_6_][Na(SO_4_)_2_]_2_[Al(H_2_O)_6_]} and the range -1/4 < *y* < 1/4, 1/4 < *y* < 3/4, *etc*. At *y*≈ 1/4 and 3/4 these layers are mutually linked *via* only two of the twelve different hydrogen bonds, O9W—H9b···O4^v^ and O13W—H13*b*···O1^vii^ (Figs. 2 and 3). These structural features agree with the preferentially tabular habit of crystals of tamarugite and their perfect cleavage on (010) (Anthony *et al.*, 2003). For structural relationships between tamarugite (NaAl(SO_4_)_2_·6H_2_O), mendozite (NaAl(SO_4_)_2_.11H_2_O; contains *trans*-Na(H_2_O)_4_(SO_4_)_2_ groups and Al(H_2_O)_6_ octahedra), and sodium alum (NaAl(SO_4_)_2_.12H_2_O; contains Na(H_2_O)_6_ and Al(H_2_O)_6_ octahedra), the reader is referred to Fang & Robinson (1972).

### 2. Experimental

Tamarugite used in this study was on a specimen of copiapite and pickeringite from "Alcaparossa, Chile", this is Mina Alcaparrosa near Cerritos Bayos, southwest of Calama, Chile, a location that furnished many well crystallized Fe^3+^ sulfate hydrates (Bandy, 1938) and is type locality for the minerals paracoquimbite, parabutlerite and the new species alcaparrosite (K_3_Ti^4+^Fe^3+^(SO_4_)_4_O(H_2_O)_2_; Kampf *et al.*, 2012).

### 3. Refinement

All hydrogen atoms were clearly visible in a difference Fourier synthesis and refined satisfactorily without restraints. For the final calculations all water molecules were idealized to have O—H = 0.80 Å and H—O—H = 108.0° and were subsequently refined as rigid groups using AFIX 6 of program *SHELXL97* (Sheldrick, 2008) with *U*_iso_(H) unrestrained. This refinement method may be considered as an approach to describe the electron density distribution of a water molecule as a fixed aspheric entity that may optionally include idealized nuclear H positions for subsequent geometric calculations.

### Crystal data

**Table:**

NaAl(SO_4_)_2_·6H_2_O	*F*(000) = 720
*M**_r_* = 350.19	*D*_x_ = 2.047 Mg m^−^^3^
Monoclinic, *P*2_1_/*a*	Mo *K*α radiation, λ = 0.71073 Å
Hall symbol: -P 2yab	Cell parameters from 8192 reflections
*a* = 7.3847 (3) Å	θ = 2.5–30.0°
*b* = 25.2814 (15) Å	µ = 0.66 mm^−^^1^
*c* = 6.1097 (3) Å	*T* = 295 K
β = 94.85 (2)°	Prism, colourless
*V* = 1136.57 (10) Å^3^	0.48 × 0.25 × 0.20 mm
*Z* = 4	

### Data collection

**Table:**

Bruker SMART CCD diffractometer	3316 independent reflections
Radiation source: fine-focus sealed tube	2826 reflections with *I* > 2σ(*I*)
Graphite monochromator	*R*_int_ = 0.029
ω and φ scans	θ_max_ = 30.1°, θ_min_ = 1.6°
Absorption correction: multi-scan (*SADABS*; Bruker, 1999)	*h* = −10→10
*T*_min_ = 0.74, *T*_max_ = 0.88	*k* = −35→35
17967 measured reflections	*l* = −8→8

### Refinement

**Table:**

Refinement on *F*^2^	Secondary atom site location: difference Fourier map
Least-squares matrix: full	Hydrogen site location: difference Fourier map
*R*[*F*^2^ > 2σ(*F*^2^)] = 0.025	H-atom parameters constrained
*wR*(*F*^2^) = 0.061	*w* = 1/[σ^2^(*F*_o_^2^) + (0.0304*P*)^2^ + 0.149*P*] where *P* = (*F*_o_^2^ + 2*F*_c_^2^)/3
*S* = 1.14	(Δ/σ)_max_ = 0.001
3316 reflections	Δρ_max_ = 0.48 e Å^−^^3^
194 parameters	Δρ_min_ = −0.36 e Å^−^^3^
0 restraints	Extinction correction: *SHELXL*, Fc^*^=kFc[1+0.001xFc^2^λ^3^/sin(2θ)]^-1/4^
Primary atom site location: structure-invariant direct methods	Extinction coefficient: 0.0154 (10)

### Special details

**Table:**

Geometry. All e.s.d.'s (except the e.s.d. in the dihedral angle between two l.s. planes) are estimated using the full covariance matrix. The cell e.s.d.'s are taken into account individually in the estimation of e.s.d.'s in distances, angles and torsion angles; correlations between e.s.d.'s in cell parameters are only used when they are defined by crystal symmetry. An approximate (isotropic) treatment of cell e.s.d.'s is used for estimating e.s.d.'s involving l.s. planes.
Refinement. Refinement of *F*^2^ against ALL reflections. The weighted *R*-factor *wR* and goodness of fit *S* are based on *F*^2^, conventional *R*-factors *R* are based on *F*, with *F* set to zero for negative *F*^2^. The threshold expression of *F*^2^ > σ(*F*^2^) is used only for calculating *R*-factors(gt) *etc*. and is not relevant to the choice of reflections for refinement. *R*-factors based on *F*^2^ are statistically about twice as large as those based on *F*, and *R*- factors based on ALL data will be even larger.

### Fractional atomic coordinates and isotropic or equivalent isotropic displacement parameters (Å^2^)

**Table:**

	*x*	*y*	*z*	*U*_iso_*/*U*_eq_	
Na	0.61674 (8)	0.05252 (2)	0.19574 (9)	0.02332 (13)	
Al	0.14270 (5)	0.145043 (15)	0.67813 (6)	0.01380 (9)	
S1	0.64309 (4)	0.183070 (12)	0.20951 (5)	0.01517 (8)	
S2	0.70759 (4)	0.020635 (12)	0.75101 (5)	0.01550 (8)	
O1	0.56130 (13)	0.22057 (4)	0.04440 (16)	0.0243 (2)	
O2	0.81572 (14)	0.16235 (5)	0.13994 (16)	0.0289 (2)	
O3	0.51612 (15)	0.13940 (4)	0.23679 (18)	0.0278 (2)	
O4	0.68102 (15)	0.21088 (4)	0.42206 (16)	0.0265 (2)	
O5	0.70649 (14)	−0.03788 (4)	0.73854 (17)	0.0236 (2)	
O6	0.75440 (14)	0.04265 (4)	0.53995 (15)	0.0264 (2)	
O7	0.52218 (13)	0.03764 (4)	0.79589 (16)	0.0223 (2)	
O8	0.83689 (14)	0.03868 (4)	0.93065 (16)	0.0253 (2)	
O9W	0.12417 (13)	0.18649 (4)	0.41967 (15)	0.01999 (19)	
H9A	0.0410	0.1799	0.3314	0.036 (5)*	
H9B	0.1394	0.2178	0.4205	0.060 (7)*	
O10W	0.06021 (14)	0.08844 (4)	0.49795 (18)	0.0246 (2)	
H10A	0.1292	0.0712	0.4331	0.040 (6)*	
H10B	−0.0271	0.0706	0.5143	0.048 (6)*	
O11W	0.38482 (13)	0.12715 (4)	0.63708 (16)	0.01946 (19)	
H11A	0.4237	0.0992	0.6801	0.042 (6)*	
H11B	0.4225	0.1328	0.5204	0.040 (6)*	
O12W	−0.09652 (13)	0.16350 (4)	0.73114 (15)	0.0214 (2)	
H12A	−0.1675	0.1793	0.6492	0.045 (6)*	
H12B	−0.1363	0.1624	0.8489	0.047 (6)*	
O13W	0.23469 (14)	0.20302 (4)	0.84445 (17)	0.0256 (2)	
H13A	0.3364	0.2044	0.9006	0.046 (6)*	
H13B	0.1744	0.2237	0.9058	0.047 (6)*	
O14W	0.13788 (16)	0.09989 (5)	0.92728 (18)	0.0325 (3)	
H14A	0.2199	0.0995	1.0216	0.073 (8)*	
H14B	0.0685	0.0768	0.9525	0.18 (2)*	

### Atomic displacement parameters (Å^2^)

**Table:**

	*U*^11^	*U*^22^	*U*^33^	*U*^12^	*U*^13^	*U*^23^
Na	0.0259 (3)	0.0258 (3)	0.0184 (3)	0.0010 (2)	0.0027 (2)	−0.0016 (2)
Al	0.01271 (17)	0.01541 (17)	0.01329 (17)	0.00230 (13)	0.00115 (12)	0.00043 (13)
S1	0.01638 (14)	0.01529 (14)	0.01387 (14)	0.00107 (10)	0.00146 (10)	0.00165 (10)
S2	0.01550 (14)	0.01615 (14)	0.01499 (14)	0.00061 (11)	0.00212 (10)	0.00103 (10)
O1	0.0220 (5)	0.0237 (5)	0.0256 (5)	−0.0051 (4)	−0.0067 (4)	0.0106 (4)
O2	0.0211 (5)	0.0454 (7)	0.0203 (5)	0.0096 (4)	0.0034 (4)	−0.0031 (4)
O3	0.0329 (6)	0.0194 (5)	0.0319 (5)	−0.0057 (4)	0.0081 (4)	0.0045 (4)
O4	0.0352 (6)	0.0237 (5)	0.0193 (5)	0.0096 (4)	−0.0046 (4)	−0.0045 (4)
O5	0.0261 (5)	0.0171 (4)	0.0283 (5)	0.0034 (4)	0.0063 (4)	−0.0015 (4)
O6	0.0256 (5)	0.0366 (6)	0.0171 (5)	−0.0108 (4)	0.0021 (4)	0.0051 (4)
O7	0.0183 (4)	0.0212 (5)	0.0278 (5)	0.0055 (4)	0.0044 (4)	0.0043 (4)
O8	0.0227 (5)	0.0335 (6)	0.0192 (5)	−0.0048 (4)	−0.0018 (4)	−0.0011 (4)
O9W	0.0221 (5)	0.0184 (5)	0.0188 (5)	−0.0015 (4)	−0.0020 (4)	0.0039 (3)
O10W	0.0216 (5)	0.0194 (5)	0.0344 (6)	−0.0046 (4)	0.0113 (4)	−0.0084 (4)
O11W	0.0171 (4)	0.0198 (5)	0.0222 (5)	0.0050 (3)	0.0056 (4)	0.0032 (4)
O12W	0.0164 (4)	0.0321 (5)	0.0160 (4)	0.0080 (4)	0.0024 (3)	0.0024 (4)
O13W	0.0164 (5)	0.0289 (5)	0.0305 (5)	0.0057 (4)	−0.0050 (4)	−0.0138 (4)
O14W	0.0282 (6)	0.0436 (7)	0.0260 (5)	0.0095 (5)	0.0041 (4)	0.0168 (5)

### Geometric parameters (Å, º)

**Table:**

Na—O6	2.2709 (11)	S2—O5	1.4814 (10)
Na—O3	2.3389 (12)	S2—O7	1.4826 (10)
Na—O8^i^	2.4153 (12)	O5—Na^ii^	2.4814 (12)
Na—O5^ii^	2.4814 (12)	O7—Na^ii^	2.5020 (11)
Na—O7^ii^	2.5020 (11)	O7—Na^iii^	2.5117 (12)
Na—O7^i^	2.5117 (12)	O8—Na^iii^	2.4153 (12)
Al—O10W	1.8755 (10)	O9W—H9A	0.80
Al—O13W	1.8776 (11)	O9W—H9B	0.80
Al—O11W	1.8816 (10)	O10W—H10A	0.80
Al—O12W	1.8816 (10)	O10W—H10B	0.80
Al—O9W	1.8904 (10)	O11W—H11A	0.80
Al—O14W	1.9054 (11)	O11W—H11B	0.80
S1—O3	1.4671 (10)	O12W—H12A	0.80
S1—O2	1.4738 (10)	O12W—H12B	0.80
S1—O1	1.4759 (10)	O13W—H13A	0.80
S1—O4	1.4825 (10)	O13W—H13B	0.80
S2—O8	1.4653 (10)	O14W—H14A	0.80
S2—O6	1.4723 (10)	O14W—H14B	0.80
			
O6—Na—O3	97.20 (4)	O2—S1—O4	108.50 (6)
O6—Na—O8^i^	109.33 (4)	O1—S1—O4	109.27 (6)
O3—Na—O8^i^	116.48 (4)	O8—S2—O6	110.61 (6)
O6—Na—O5^ii^	101.25 (4)	O8—S2—O5	110.48 (6)
O3—Na—O5^ii^	78.70 (4)	O6—S2—O5	109.46 (6)
O8^i^—Na—O5^ii^	142.95 (4)	O8—S2—O7	108.94 (6)
O6—Na—O7^ii^	91.90 (4)	O6—S2—O7	109.95 (6)
O3—Na—O7^ii^	135.96 (4)	O5—S2—O7	107.34 (6)
O8^i^—Na—O7^ii^	100.49 (4)	S1—O3—Na	118.86 (6)
O5^ii^—Na—O7^ii^	57.26 (3)	S2—O5—Na^ii^	98.15 (5)
O6—Na—O7^i^	161.83 (5)	S2—O6—Na	137.12 (6)
O3—Na—O7^i^	100.38 (4)	S2—O7—Na^ii^	97.25 (5)
O8^i^—Na—O7^i^	58.24 (3)	S2—O7—Na^iii^	92.19 (5)
O5^ii^—Na—O7^i^	86.73 (4)	Na^ii^—O7—Na^iii^	101.38 (4)
O7^ii^—Na—O7^i^	78.62 (4)	S2—O8—Na^iii^	96.57 (5)
O10W—Al—O13W	176.35 (5)	Al—O9W—H9A	116.5
O10W—Al—O11W	90.20 (5)	Al—O9W—H9B	122.9
O13W—Al—O11W	87.45 (5)	H9A—O9W—H9B	108.0
O10W—Al—O12W	91.56 (5)	Al—O10W—H10A	121.1
O13W—Al—O12W	90.87 (5)	Al—O10W—H10B	125.6
O11W—Al—O12W	177.69 (5)	H10A—O10W—H10B	108.0
O10W—Al—O9W	86.31 (5)	Al—O11W—H11A	119.2
O13W—Al—O9W	90.96 (5)	Al—O11W—H11B	118.8
O11W—Al—O9W	91.40 (5)	H11A—O11W—H11B	108.0
O12W—Al—O9W	90.21 (5)	Al—O12W—H12A	126.2
O10W—Al—O14W	89.13 (6)	Al—O12W—H12B	124.6
O13W—Al—O14W	93.76 (6)	H12A—O12W—H12B	108.0
O11W—Al—O14W	92.56 (5)	Al—O13W—H13A	123.4
O12W—Al—O14W	85.98 (5)	Al—O13W—H13B	125.0
O9W—Al—O14W	173.97 (5)	H13A—O13W—H13B	108.0
O3—S1—O2	110.00 (7)	Al—O14W—H14A	121.3
O3—S1—O1	109.42 (6)	Al—O14W—H14B	130.3
O2—S1—O1	110.20 (6)	H14A—O14W—H14B	108.0
O3—S1—O4	109.43 (6)		

Symmetry codes: (i) *x*, *y*, *z*−1; (ii) −*x*+1, −*y*, −*z*+1; (iii) *x*, *y*, *z*+1.

### Hydrogen-bond geometry (Å, º)

**Table:**

*D*—H···*A*	*D*—H	H···*A*	*D*···*A*	*D*—H···*A*
O9*W*—H9*A*···O2^iv^	0.80	2.00	2.7971 (15)	173
O9*W*—H9*B*···O4^v^	0.80	1.83	2.6282 (14)	178
O10*W*—H10*A*···O5^ii^	0.80	1.87	2.6665 (14)	173
O10*W*—H10*B*···O6^iv^	0.80	1.78	2.5697 (14)	169
O11*W*—H11*A*···O7	0.80	1.83	2.6315 (14)	176
O11*W*—H11*B*···O3	0.80	1.93	2.7236 (14)	175
O12*W*—H12*A*···O4^iv^	0.80	1.89	2.6787 (14)	171
O12*W*—H12*B*···O2^vi^	0.80	1.84	2.6330 (14)	169
O13*W*—H13*A*···O1^iii^	0.80	1.86	2.6474 (14)	169
O13*W*—H13*B*···O1^vii^	0.80	1.88	2.6698 (14)	171
O14*W*—H14*A*···O5^viii^	0.80	2.18	2.7478 (15)	129
O14*W*—H14*B*···O8^iv^	0.80	1.96	2.7100 (17)	156

Symmetry codes: (ii) −*x*+1, −*y*, −*z*+1; (iii) *x*, *y*, *z*+1; (iv) *x*−1, *y*, *z*; (v) *x*−1/2, −*y*+1/2, *z*; (vi) *x*−1, *y*, *z*+1; (vii) *x*−1/2, −*y*+1/2, *z*+1; (viii) −*x*+1, −*y*, −*z*+2.
